# Digital health literacy and associated factors in older adult patients with chronic obstructive pulmonary disease: a latent profile analysis

**DOI:** 10.3389/fpubh.2026.1880553

**Published:** 2026-08-17

**Authors:** Mengyan Wu, Zhiqing Zhou, Miao Zhang, Huan Qiu, Shijun Huang, Xingsheng Wang, Nan Mo, Yurong Wang, Yinyin Tang, Xiyao Yang, Manzhen Bao

**Affiliations:** 1 School of Nursing, Anhui Medical University, Hefei, Anhui, China; 2 Department of Nursing, The Second Affiliated Hospital of Anhui Medical University, Hefei, Anhui, China; 3 Anhui Medical University, Taikang Health Industry Research Institute, Hefei, Anhui, China

**Keywords:** associated factors, chronic obstructive pulmonary disease, digital health literacy, latent profile analysis, older adult patients

## Abstract

**Objective:**

To identify latent profiles of digital health literacy (DHL) among older adult patients with chronic obstructive pulmonary disease (COPD) and to examine the factors associated with profile membership.

**Methods:**

Between December 2025 and February 2026, a total of 575 older adult patients with COPD were assessed for eligibility from both the inpatient wards and the respiratory medicine outpatient clinic of a Grade A tertiary hospital in Anhui Province. The study site was selected using a convenience sampling method. To minimize selection bias and ensure representativeness within this setting, all eligible patients who presented during the study period were consecutively recruited. Of these, 550 provided valid responses and were included in the final analysis. Data collection was conducted using a set of structured questionnaires, which comprised the following instruments: a General Information Questionnaire, the digital health literacy (DHL), the COPD Self-Efficacy Scale (CSES), the Technophobia Scale (TS), and the Perceived Social Support Scale (PSSS). To identify distinct subgroups based on digital health literacy profiles, latent profile analysis (LPA) was performed using Mplus 8.3 software. Univariate and multinomial logistic regression analyses were conducted using SPSS 27.0 software to identify factors associated with profile membership.

**Results:**

Three DHL profiles were identified: low literacy-restricted basic skills type (37.1%), moderate literacy-guided learning dependent type (28.9%), and high literacy-independent efficient type (34.0%). Factors significantly associated with profile membership included education level, income, health information seeking intention, self-efficacy, technophobia, and social support.

**Conclusion:**

Overall DHL levels were generally low among older adult patients with COPD, with significant variation across demographic subgroups. Personalized interventions tailored to patients' specific profile characteristics and associated factors may represent a promising strategy for enhancing DHL in this patient population. However, as these intervention strategies are derived from correlational profile data, their effectiveness requires prospective validation in future interventional studies.

## 1 Introduction

Chronic Obstructive Pulmonary Disease (COPD) is a progressive and irreversible condition characterized by persistent respiratory symptoms and airflow limitation. Its prevalence rises markedly with advancing age-a trend exacerbated by global population aging (1). Among individuals aged 60 years and older, the prevalence exceeds 27%, and this figure increases to approximately 35.5% in those aged 70 years and above (2–4). As a major respiratory disease, COPD is characterized by high prevalence, a heavy disease burden, and high readmission rates. For example, a systematic review indicated that the 30-day and 90-day all-cause readmission rates for patients with COPD were 18 % and 31 %, respectively. COPD severely impairs the quality of life and prognosis of older adult patients, placing sustained pressure on healthcare systems and families (5, 6). Therefore, it is particularly urgent to explore effective ways to improve health management capacity in this population.

Effective long-term disease control for patients with COPD relies heavily on their ability to perform consistent self-management (7). However, many older adult patients face significant barriers in this regard, highlighting an urgent need for innovative strategies to enhance their health management capacity (8).The rapid evolution of digital health technologies is opening up new avenues for transforming self-management strategies in COPD care (9). Digital health literacy (DHL) refers to an individual's ability to access, evaluate, interact with, and apply digital health information to maintain or improve their health (10). Existing research indicates that self-efficacy, technophobia, and social support are significant factors associated with digital health literacy (11).

The health ecology theory posits that individuals' health behaviors and health outcomes are jointly shaped by the interplay of multilevel factors, including individual, behavioral, interpersonal network, living conditions, and policy environment dimensions. Based on this framework, the individual level encompasses variables such as gender, age, education level, disease course of COPD, hospitalizations within the past year, and gold classification; the behavioral and psychological characteristics level includes smoking status, internet use frequency, health information seeking intention, query skill, health software usage, as well as self-efficacy and technophobia; the interpersonal network level is assessed by marital status and the social support; the living conditions level incorporates place of residence and monthly income; and the policy environment level is represented by type of medical insurance. This study aims to explore how the aforementioned multilevel factors are synergistically are associated with the heterogeneity of DHL among older adult patients with COPD.

It is noteworthy that DHL within this cohort is not uniform, but instead exhibits substantial internal heterogeneity. To delve deeper into the diverse patterns of competency characteristics and precisely identify the factors associated among different characteristic groups, this study employs Latent profile analysis (LPA). As a person-centered analytical method, LPA classifies individuals based on shared response patterns, thereby identifying latent subgroups and revealing population heterogeneity with greater precision (12, 13). Therefore, this study employs latent profile analysis to explore the DHL profiles in older adult patients with COPD. Based on health ecology theory, this study analyzes the factors associated with each profile, thereby providing a basis for developing personalized intervention approaches.

## 2 Materials and methods

### 2.1 Participants

This study used a convenience sampling method to select the study site. Participants were consecutively recruited from the respiratory medicine outpatient clinic and inpatient wards of a Grade A tertiary general hospital in Anhui Province between December 2025 and February 2026. The inclusion criteria were defined as follows: (1) diagnosis of COPD according to the GOLD guidelines (14); (2) age ≥60 years; (3) voluntary participation in this study and provision of written informed consent. Conversely, exclusion criteria comprised: (1) concomitant severe organic diseases of the heart, liver, kidneys, etc.; (2) presence of psychiatric disorders; (3) presence of severe visual or auditory impairment.

**Figure 1:**
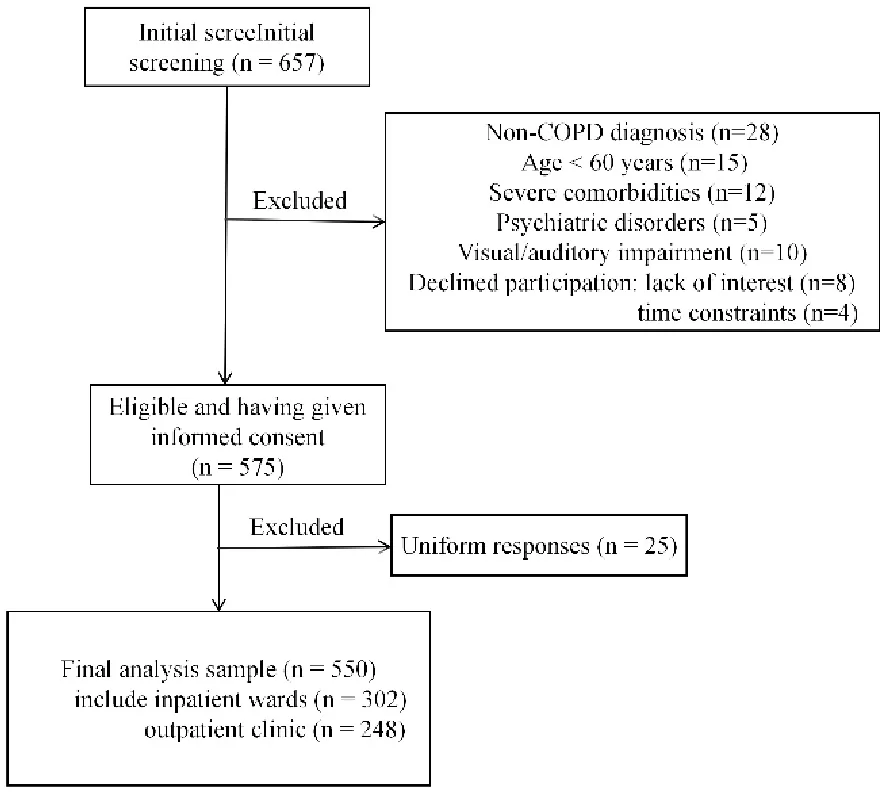
Participant flow diagram.

A total of 657 patients were initially screened during the study period. Of these, 70 patients were excluded for not meeting the GOLD diagnostic criteria (*n* = 28), age < 60 years (*n* = 15), severe comorbid organic diseases of the heart, liver, or kidneys (*n* = 12), psychiatric disorders (*n* = 5), and severe visual or auditory impairment (*n* = 10). In addition, 12 patients declined to participate (lack of interest, *n* = 8; time constraints, *n* = 4). After these exclusions, all 575 eligible patients provided consent and completed the questionnaire survey. Following data collection, 25 questionnaires were excluded from the final analysis because every item was answered identically, leaving a total of 550 valid questionnaires for the final analysis. Specifically, 302 valid questionnaires were obtained from inpatient wards and 248 from outpatient departments. A detailed participant flow diagram is presented in Figure 1.

The required sample size was calculated using G^*^Power 3.1 for regression analysis, assuming a medium effect size f^2^ = 0.15, α = 0.05, and a statistical power of 0.95 (15). With up to 33 predictor variables anticipated and accounting for an estimated 20% non-response rate, the calculated target sample size was 338 cases. Moreover, according to Nylund-Gibson (16), a sample of at least 300 is recommended for LPA to ensure model convergence and to detect relatively small latent classes. To meet these requirements and allow for potential invalid responses, we recruited 575 older adult patients with COPD, all of whom completed the questionnaire survey. After excluding 25 invalid questionnaires (due to identical responses across all items), 550 valid participants were included in the final analysis. This final sample exceeded the minimum needed for both regression and LPA. This study was reviewed and approved by the Ethics Committee of Anhui Medical University (Approval No: 81250962).

### 2.2 Measures

#### 2.2.1 General information questionnaire

The questionnaire was developed by the research team based on a literature review and consultation with clinical specialists, comprising four sections: (1) socio-demographic characteristics: gender, age, marital status, place of residence, educational level, monthly income, medical insurance type and smoking history; (2) disease-related information: disease course of COPD (years), hospitalizations within the past year, and GOLD classification; (3) Internet usage characteristics: including frequency of internet use, health information seeking intention, general online query skill, and health software usage. These items measure behavioral engagement, motivational tendency and basic digital practice experience, which are conceptually distinct from the competency-focused DHL construct. They were included as potential factors associated with DHL heterogeneity guided by the health ecology theoretical framework.

#### 2.2.2 Digital health literacy

The original instrument was constructed by Van der Vaart (17), and later adapted into Chinese and validated by Zhao (18). This instrument consists of two distinct sections: a self-assessment component and an objective assessment component. The self-assessment section consists of 21 items across seven dimensions (3 items per dimension): operational skills, navigation skills, information searching, determining relevance, evaluating reliability, adding content, and protecting privacy. Each item is rated on a 5-point Likert scale (0 = “very difficult/never” to 4 = “very easy/always”). The score for each dimension is the average of the item scores within that dimension, with a possible range of 0 to 4. The total score calculated as the sum of all 21 item scores, ranges from 0 to 84; higher scores indicate greater DHL. The objective assessment section is designed to measure practical skills through seven items, with one item per dimension. This procedure employs a dichotomous scoring system, wherein accurate responses are assigned a value of 1, while erroneous or ambiguous replies are allotted a value of 0. In the Chinese validation study, the self-assessment scale demonstrated good reliability, with a total Cronbach's α of 0.929 and dimension-specific coefficients ranging from 0.769 to 0.920 (18). In the present sample (*n* = 550), we also calculated the internal consistency of the self-assessment scale and its seven dimensions using the collected data. The total Cronbach's α was 0.931, and the dimension-specific coefficients were as follows: operational skills (α = 0.808), navigation skills (α = 0.765), information searching (α = 0.897), determining relevance (α = 0.915), evaluating reliability (α = 0.900), adding content (α = 0.902), and protecting privacy (α = 0.845). These estimates were consistent with those reported in the validation study. Of note, the exclusion of the objective assessment component was not prespecified in the original study protocol. It was initially planned for inclusion in the data collection. However, during the actual administration in our older adult COPD population, we observed substantial feasibility challenges, including difficulty in understanding complex task instructions, prolonged completion time, and poor participant cooperation. In addition, the Chinese validation study had already reported low reliability for this objective component (Cronbach's α = 0.565) (18). Given these methodological concerns, the research team made a *post-hoc* decision at the data entry and preliminary analysis stage to exclude this component from the final analysis. This decision is consistent with the approach recommended by the original scale developers when using the DHL instrument in older populations (17). In this study, only the self-assessment component was used for the final analysis.

#### 2.2.3 COPD self-efficacy scale

The COPD Self-Efficacy Scale (CSES), initially created by Wigal J K. for assessing self-efficacy in patients with COPD (19), subsequently underwent translation and cross-cultural adaptation for the Chinese population through the efforts of a bilingual nursing scholar (20). This adapted instrument consists of 31 items across five dimensions: negative affect, intense emotional arousal, physical exertion, weather/environment, and behavioral risk factors. The total score ranges from 31 to 155 points. Higher scores reflect greater levels of patient self-efficacy. In this study, the scale Cronbach's α was 0.88.

#### 2.2.4 Technophobia scale

This study employed the Technophobia Scale (TS) to assess technophobia, originally developed by Khasawneh (21) and later adapted into Chinese by Sun (22). The scale consists of 13 items across three dimensions: techno-anxiety, techno-paranoia, and privacy concerns. Item responses were obtained using a five-point Likert scale, anchored by 1 (representing strong disagreement) and 5 (representing strong agreement). Summing these ratings produced total scores between 13 and 65, with elevated scores corresponding to heightened levels of technophobia. In this study, the Cronbach's α was 0.911.

#### 2.2.5 Perceived social support scale

The level of support from both familial and extra-familial sources was evaluated using the Perceived Social Support Scale (PSSS). Originally developed by Zimet (23), the 12-item instrument was subsequently adapted and validated in Chinese by Jiang (24). Three dimensions-family, friend, and other support-are assessed, with total scores ranging from 12 to 84, where higher scores reflect stronger perceived support. In this study, the Cronbach's α was 0.90.

### 2.3 Data collection

Researchers rigorously screened older adult patients with COPD to ensure compliance with inclusion and exclusion criteria. Prior to participation, individuals were thoroughly briefed on the research aims, protocols, and confidentiality measures, after which written informed consent was obtained from all participants. All questionnaires were administered using paper-based forms. Participants were asked to complete the questionnaires independently, with researchers providing clarification only upon request to avoid interference. In cases where independent completion was not feasible, the questionnaire was administered orally in a one-to-one setting. Throughout this process, leading questions was avoided, and responses were strictly recorded based on the participants' own accounts. Completed questionnaires were collected and verified immediately. All data were entered independently by two individuals, with 10.0% of entries randomly cross-checked for accuracy.

### 2.4 Statistical analysis

Following repeated verification and double data entry, latent variable modeling was performed using Mplus 8.3 software. For the LPA, the seven dimension means of the DHL self-assessment scale were entered as continuous indicator variables. These dimensions included operational skills, navigation skills, information searching, determining relevance, evaluating reliability, adding content, and protecting privacy. LPA was conducted using robust maximum likelihood estimation (MLR) to obtain robust standard errors. To avoid convergence to local maxima, following relevant guidelines, we specified 200 random sets of starting values and performed 50 final-stage optimizations; the convergence criterion was set such that the change in log-likelihood was less than 1 × 10^−6^ and the maximum absolute change in parameter estimates was less than 1 × 10^−6^. All models converged successfully (25). The final analytic sample (*n* = 550) contained no missing data. To evaluate the assumption of local independence, we examined residual correlations among the seven dimensions of DHL within each latent profile. Taking a correlated LPA as a reference, all residual correlation coefficients were below 0.05, indicating that the local independence assumption was reasonably satisfied (26). We fitted models with one to four latent profiles, and the optimal model was determined through a comprehensive evaluation of fit indices and clinical interpretability. The model assessment criteria included: (1) the Akaike Information Criterion (AIC), Bayesian Information Criterion (BIC), and adjusted Bayesian Information Criterion (aBIC), with lower values indicating better fit; (2) Entropy (range 0–1), where values closer to 1 reflect higher classification accuracy; and (3) the Lo-Mendell-Rubin likelihood ratio test (LMR) and Bootstrap Likelihood Ratio Test (BLRT), with *P* < 0.05 supporting the superiority of the K-class model over the K-1 class model.

Subsequently, all statistical computations were conducted utilizing SPSS 27.0. Prior to the computation of descriptive statistics, normality was assessed for all continuous variables. Normality of continuous variables was tested using the Kolmogorov-Smirnov test. Normally distributed data were expressed as mean ± standard deviation (SD), with inter-group differences assessed via one-way analysis of variance (ANOVA). For categorical data, descriptive statistics were reported as frequencies and percentages (*n*%). Inter-group differences were assessed using the Chi-square test or Fisher's exact test. To further investigate the factors associated with the identified categories, variables that demonstrated statistical significance in univariate analyses were subsequently incorporated into a multinomial logistic regression model. Predictor selection was guided by the health ecology theoretical framework and a backward stepwise approach, which retained only variables with independent, significant effects after controlling for covariates. Notably, the backward stepwise selection method has inherent methodological limitations: it may inflate Type I error due to repeated hypothesis testing, lead to overestimated regression coefficients and underestimated standard errors, and the final model may be overly dependent on the characteristics of the current sample, resulting in potential overfitting. To address these limitations and verify the robustness of core findings, we additionally constructed a theoretically specified full model as a sensitivity analysis. In this full model, all variables with statistical significance in univariate analysis were simultaneously included in the multinomial logistic regression without stepwise selection, to examine whether the direction and significance of core predictors remained stable after controlling for all covariates simultaneously.

Prior to building the final model, several diagnostic procedures were conducted to ensure robustness. To assess multicollinearity, variance inflation factors (VIF) were calculated for all predictors. The linearity of the logit for continuous variables (CSES, TS, PSSS) was checked using the Box-Tidwell test. Influential observations were detected using Cook's distance and DFBeta values. Given the sparse data in some cells, which could lead to quasi-complete separation in conventional maximum likelihood estimation, Firth's penalized likelihood estimation was applied as a sensitivity analysis to confirm the stability of the odds ratios (ORs). The Firth's penalized logistic regression analyses were performed in R (version 4.4.3) using the logistf package. This method introduces a penalty term derived from Jeffreys' invariant prior into the standard log-likelihood function. In the logistf package, model parameters are estimated via a modified Newton–Raphson algorithm. Since the outcome variable contained three latent DHL profiles, three separate binary Firth's penalized logistic regressions were conducted corresponding to the three pairwise comparisons in the primary multinomial analysis (C2 vs. C1, C3 vs. C1, C3 vs. C2). Each model included the same set of predictors retained in the final backward stepwise multinomial logistic regression. The reference category settings were fully consistent with the primary analysis: for profile comparisons, C1 served as the reference group for C2 vs. C1 and C3 vs. C1, while C2 served as the reference for C3 vs. C2; for independent variables, the reference categories were “high school and above” for education level, “≥5,000 RMB” for monthly income, and “willing” for health information seeking intention, with ORs for continuous variables calculated per 10-point increase. Model goodness-of-fit was evaluated using the Likelihood Ratio Test, Nagelkerke R^2^, and classification accuracy. Furthermore, to explicitly address the classification uncertainty inherent in latent profile analysis, an additional sensitivity analysis was performed using the R3STEP three-step procedure in Mplus 8.3 (27). This method follows three sequential steps: (1) estimation of the latent profile measurement model based on DHL dimension indicators; (2) assignment of individuals to their most likely latent class while quantifying classification error; (3) estimation of associations between covariates and class membership, with formal adjustment for the measurement error derived from Step 2. The results of this error-adjusted approach were directly compared with those of the primary classify-analyze approach to verify the stability of covariate effect estimates. All statistical tests were conducted with *P* < 0.05 as the threshold for statistical significance.

## 3 Results

### 3.1 Common method bias test

Given that this study relied exclusively on questionnaire surveys for data collection, Harman's one-factor test was employed to evaluate potential common method bias. The analysis extracted ten factors with eigenvalues exceeding 1. The largest single factor accounted for 26.436% of the total variance, a figure that falls below the 40% threshold commonly applied in such diagnostics (28). Thus, common method bias was not considered a substantial threat in the present study.

### 3.2 General information of the participants

Figure 1 illustrates the participant recruitment and screening process. A total of 657 patients were initially screened. After excluding 70 patients who did not meet the inclusion criteria and 12 who declined to participate, 575 eligible patients were enrolled and completed the survey. Following the exclusion of 25 questionnaires with uniform responses, 550 valid responses were included in the final analysis, yielding a valid response rate of 95.65%. The study sample comprised 550 older adult patients diagnosed with COPD, with a mean age of 72.65 years (SD = 6.131). The sample included 336 males (61.1%) and 214 females (38.9%). Further sociodemographic and clinical characteristics are summarized in Table 1.

**Table 1:** General data and univariate analysis of potential profiles of digital health literacy characteristics in older adult patients with COPD.

Variable	Total	C1	C2	C3	*χ^2^/F*	*P*
	(*n* = 550)	(*n* = 204)	(*n* = 159)	(*n* = 187)		
Age					120.530^a^	**< 0.001**
60~69	165 (30.0%)	25 (12.3%)	32 (20.1%)	108 (57.8%)		
70~79	336 (61.1%)	145 (71.1%)	119 (74.8%)	72 (38.5%)		
≥80	49 (8.9%)	34 (16.7%)	8 (5.0%)	7 (3.7%)		
Gender					2.131^a^	0.345
Male	336 (61.1%)	124 (60.8%)	104 (65.4%)	108 (57.8%)		
Female	214 (38.9%)	80 (39.2%)	55 (34.6%)	79 (42.2%)		
Marital status					10.446^a^	**0.005**
Married	509 (92.5%)	180 (88.2%)	148 (93.1%)	181 (96.8%)		
Unmarried/divorced/widowed	41 (7.5%)	24 (11.8%)	11 (6.9%)	6 (3.2%)		
Place of residence					26.377^a^	**< 0.001**
City	309 (56.2%)	143 (70.1%)	72 (45.3%)	94 (50.3%)		
Rural area	241 (43.8%)	61 (29.9%)	87 (54.7%)	93 (49.7%)		
Education level					343.287^a^	**< 0.001**
Elementary school and under	320 (58.2%)	190 (93.1%)	121 (76.1%)	9 (4.8%)		
Middle school	132 (24.0%)	11 (5.4%)	22 (13.8%)	99 (52.9%)		
High school and above	98 (17.8%)	3 (1.5%)	16 (10.1%)	79 (42.2%)		
Monthly income (RMB)					305.012^a^	**< 0.001**
< 1,000	255 (46.4%)	174 (85.3%)	73 (45.9%)	8 (4.3%)		
1,000~2,999	145 (26.4%)	18 (8.8%)	64 (40.3%)	63 (33.7%)		
3,000~4,999	106 (19.3%)	8 (3.9%)	16 (10.1%)	82 (43.9%)		
≥5,000	44 (8.0%)	4 (2.0%)	6 (3.8%)	34 (18.2%)		
Medical insurance type					47.843^a^	**< 0.001**
Resident insurance	483 (87.8%)	196 (96.1%)	144 (90.6%)	143 (76.5%)		
Employee insurance	55 (10.0%)	3 (1.5%)	11 (6.9%)	41 (21.9%)		
Commercial insurance or other	12 (2.2%)	5 (2.5%)	4 (2.5%)	3 (1.6%)		
Smoking					17.913^a^	**< 0.001**
Yes	22 (4.0%)	1 (0.5%)	5 (3.1%)	16 (8.6%)		
No	128 (23.3%)	47 (23.0%)	35 (22.0%)	46 (24.6%)		
Quit smoking	400 (72.7%)	156 (76.5%)	119 (74.8%)	125 (66.8%)		
Disease course of COPD (years)					72.454^a^	**< 0.001**
< 5	135 (24.5%)	21 (10.3%)	37 (23.3%)	77 (41.2%)		
5~10	266 (48.4%)	96 (47.1%)	83 (52.2%)	87 (46.5%)		
>10	149 (27.1%)	87 (42.6%)	39 (24.5%)	23 (12.3%)		
Hospitalizations within the past year					103.192^a^	**< 0.001**
0	55 (10.0%)	4 (2.0%)	6 (3.8%)	45 (24.1%)		
1	125 (22.7%)	23 (11.3%)	42 (26.4%)	60 (32.1%)		
≥2	370 (67.3%)	177 (86.8%)	111 (69.8%)	82 (43.9%)		
GOLD classification					181.322^a^	**< 0.001**
I	12 (2.2%)	2 (1.0%)	2 (1.3%)	8 (4.3%)		
II	219 (39.8%)	35 (17.2%)	55 (34.6%)	129 (69.0%)		
III	246 (44.7%)	102 (50.0%)	94 (59.1%)	50 (26.7%)		
IV	73 (13.3%)	65 (31.9%)	8 (5.0%)	0 (0.0%)		
Frequency of Internet use					612.949^a^	**< 0.001**
Seldom	192 (34.9%)	178 (87.3%)	14 (8.8%)	0 (0.0%)		
Sometimes	107 (19.5%)	19 (9.3%)	88 (55.3%)	0 (0.0%)		
Always	251 (45.6%)	7 (3.4%)	57 (35.8%)	187 (100.0%)		
Health information seeking intention					358.059^a^	**< 0.001**
Willing	253 (46.0%)	11 (5.4%)	56 (35.2%)	186 (99.5%)		
Unwilling	297 (54.0%)	193 (94.6%)	103 (64.8%)	1 (0.5%)		
Query skill					695.343^a^	**< 0.001**
Unable to complete	194 (35.3%)	176 (86.3%)	18 (11.3%)	0 (0.0%)		
Can complete with assistance	146 (26.5%)	24 (11.8%)	117 (73.6%)	5 (2.7%)		
Can complete independently	210 (38.2%)	4 (2.0%)	24 (15.1%)	182 (97.3%)		
Health software usage					52.975^a^	**< 0.001**
Yes	26 (4.7%)	0 (0.0%)	0 (0.0%)	26 (13.9%)		
No	524 (95.3%)	204 (100.0%)	159 (100.0%)	161 (86.1%)		
DHL	27.21 ± 19.96	6.71 ± 3.08	23.81 ± 4.30	52.47 ± 6.12	4,465.074^b^	**< 0.001**
CSES	57.94 ± 23.16	36.99 ± 3.69	51.21 ± 12.40	86.53 ± 9.78	2,154.387^b^	**< 0.001**
TS	37.52 ± 15.84	51.00 ± 3.43	42.66 ± 8.24	18.44 ± 9.05	278.197^b^	**< 0.001**
PSSS	43.30 ± 22.55	25.39 ± 5.88	32.97 ± 12.62	71.62 ± 8.86	307.723^b^	**< 0.001**

Bold, *P* < 0.05; a, χ^2^; b, F; C1, low literacy-restricted basic skills type; C2, moderate literacy-guided learning dependent type; C3, high literacy-independent and efficient type. DHL, digital health literacy; CSES, the COPD Self-Efficacy Scale; TS, Technophobia Scale; PSSS, Perceived Social Support Scale.

### 3.3 Latent profile analysis of digital health literacy in older adult patients with COPD

LPA was performed using the seven dimensions of the DHL rating scale as manifest indicators. Models specifying one to four latent profiles were estimated sequentially, with fit indices summarized in Table 2. Although the AIC, BIC, and aBIC values continued to decrease from the three-profile to the four-profile model, model selection was based on a comprehensive evaluation of multiple criteria. The four-profile solution showed non-significant BLRT (*P* = 0.105) and LMR (*P* = 0.100) tests, indicating no statistically meaningful improvement over the three-profile model. Moreover, the four-profile model contained a very small class (13.8%), raising concerns about stability and potential overextraction. The three-profile model demonstrated superior classification accuracy (Entropy = 0.995 vs. 0.974), more balanced class proportions, greater clinical interpretability with distinct and meaningful profiles, and better parsimony. Classification quality was further confirmed by average posterior probabilities of 0.992 for Class 1, 0.995 for Class 2, and 0.998 for Class 3, all substantially above the recommended threshold of 0.80 (29). The overall classification error rate was 0.5%, indicating high discriminatory power. Therefore, the three-profile model was selected for further interpretation in this study.

**Table 2:** Model fit indices for the compared latent profiles (*n* = 550).

Modle	Log (L)	AIC	BIC	aBIC	Entropy	BLRT	LMR	Class probability
1	−9,667.622	19,363.244	19,423.582	19,379.140				
2	−7,438.902	14,921.805	15,016.623	14,946.786	0.992	< 0.001	< 0.001	0.647/0.353
3	−6,502.797	13,065.594	13,194.891	13,099.658	0.995	< 0.001	< 0.001	0.371/0.289/0.340
4	−6,133.200	12,342.401	12,506.178	12,385.549	0.974	0.105	0.100	0.373/0.138/0.213/0.276

*P* < 0.05 was considered statistically significant. AIC, Akaike information criterion; BIC, Bayesian information criterion; aBIC, sample-size-adjusted Bayesian information criterion; Entropy, information entropy; LMR, Lo-Mendell-Rubin Adjusted Likelihood Ratio Test; BLRT, the Bootstrap Likelihood Ratio Test.

**Figure 2:**
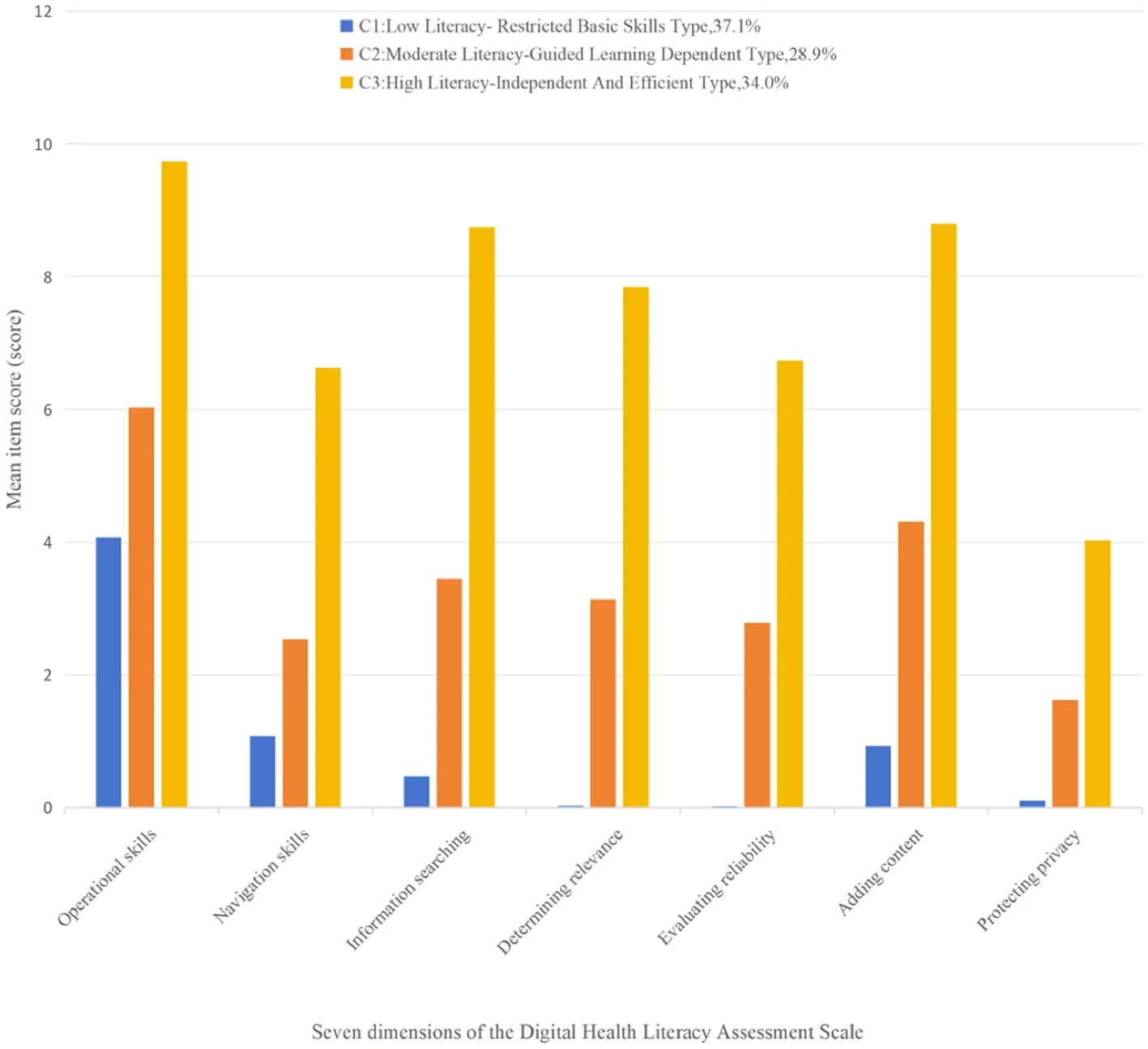
The characteristic distribution of three latent profiles of digital health literacy in older adult patients with COPD.

Figure 2 illustrates the score distributions across seven dimensions of DHL among the three identified latent categories of older adult patients with COPD. C1: comprising 37.1% (*n* = 204) of the sample, was characterized by lower scores across all dimensions and was therefore designated as the low literacy-restricted basic skills type. C2: representing 28.9% (*n* = 159) of the sample, exhibited moderate proficiency in foundational operational skills. However, relatively lower competence in navigation and privacy protection was observed, indicating a reliance on external guidance or structured support. This group was consequently defined as moderate literacy-guided learning dependent type. C3: demonstrated uniformly high scores across all literacy dimensions, with particular strength observed in foundational skills and content creation. Accounting for 34.0% (*n* = 187) of the total sample, this category was classified as high literacy-independent and efficient type.

### 3.4 Univariate analysis of latent classes of digital health literacy in older adult patients with COPD

Univariate analysis identified statistically significant differences (*P* < 0.05) between the three DHL categories across the following variables: age, marital status, place of residence, educational level, monthly income, type of medical insurance, smoking status, disease course of COPD, hospitalization, Gold classification, frequency of internet use, health information seeking intention, query skill, health software usage, self-efficacy, technophobia, and perceived social support. Detailed results are presented in Table 1.

### 3.5 Multivariate analysis of factors associated with digital health literacy profiles in older adult patients with COPD

Multinomial logistic regression analysis was employed to examine factors associated with the three latent categories of DHL among older adult patients with COPD. Variables showing significant differences in univariate analyses were included as independent variables. All categorical independent variables were transformed into dummy variables, with specific assignments detailed in Table 3. The results of the regression analysis are presented in Table 4. Compared with the low literacy–restricted basic skills type (C1, reference group), patients with an education level of primary school or below were significantly less likely to be classified into the moderate literacy–guided learning dependent type (C2) and the high literacy–independent efficient type (C3) (OR = 0.068, 95% CI: 0.007–0.649; OR = 0.088, 95% CI: 0.009–0.835, respectively). Patients with a monthly income of < 1,000 yuan also had significantly lower odds of being in C2 and C3 (OR = 0.036, 95% CI: 0.011–0.115; OR = 0.011, 95% CI: 0.001–0.147, respectively). Moreover, patients who were unwilling to actively seek health information were significantly less likely to be in C2 and C3 compared with those in C1 (OR = 0.239, 95% CI: 0.116–0.492; OR = 0.092, 95% CI: 0.030–0.285, respectively). With respect to continuous variables (calculated per 10-point increase), for each 10-point increase in CSES, the odds of belonging to the C2 and C3 groups, relative to C1, were 11.256 times greater (95% CI: 4.849–26.147) and 28.919 times greater (95% CI: 8.951–93.299), respectively. For each 10-point increase in TS, the odds of being in C2 decreased (OR = 0.061, 95% CI: 0.021–0.171), and the odds of being in C3 decreased (OR = 0.072, 95% CI: 0.015–0.356). For each 10-point increase in PSSS, the odds of being in C2 and C3 were 2.918 times greater (95% CI: 1.480–5.737) and 3.975 times greater (95% CI: 1.951–8.050), respectively.

**Table 3:** Assignment table for multifactorial analysis of latent classes of digital health literacy in older adult patients with COPD.

Variables	Assignment of variables
Age	60~69 (reference)
	X1: 70~79 = 1, ≥80 = 0
	X2: 70~79 = 0, ≥80 = 1
Marital status	Married = 0, Unmarried/divorced/widowed = 1
Place of residence	City = 0, Rural area = 1
Education level	High school and above (reference) X1: Elementary school and under = 1, Middle school = 0
	X2: Elementary school and under = 0, Middle school = 1
Monthly income (RMB)	≥5,000 (reference)
	X1: < 1,000 = 1, 1,000~2,999 = 0, 3,000~4,999 = 0
	X2: < 1,000 = 0, 1,000~2,999 = 1, 3,000~4,999 = 0
	X3: < 1,000 = 0, 1,000~2,999 = 0, 3,000~4,999 = 1
Medical insurance type	Commercial insurance or other (reference)
	X1: Resident insurance = 1, Employee insurance = 0
	X2: Resident insurance = 0, Employee insurance = 1
Smoking	Quit smoking (reference)
	X1: Yes = 1, No = 0
	X2: Yes = 0, No = 1
Disease course of COPD (years)	>10 (reference)
	X1: < 5 = 1, 5~10 = 0
	X2: < 5 = 0, 5~10 = 1
Hospitalizations within the past year	≥2 (reference)
	X1: 0 = 1, 1 = 0
	X2: 0 = 0, 1 = 1
GOLD classification	IV (reference)
	X1: I = 1, II = 0, III = 0
	X2: I = 0, II = 1, III = 0
	X3: I = 0, II = 0, III = 1
Frequency of internet use	Always (reference)
	X1: Seldom = 1, Sometimes = 0
	X2: Seldom = 0, Sometimes = 1
Health information seeking intention	Willing = 0, Unwilling = 1
Query skill	Can complete independently (reference)
	X1: Unable to complete = 1, Can complete with assistance = 0
	X2: Unable to complete = 0, Can complete with assistance = 1
Health software usage	Yes = 0, No = 1
COPD self-efficacy scale	Substitution with the original value
Technophobia scale	Substitution with the original value
Perceived social support scale	Substitution with the original value

**Table 4:** Multifactorial analysis of latent classes of digital health literacy in older adult patients with COPD.

Variables	C2 vs. C1 (ref: C1)		C3 vs. C1 (ref: C1)		C3 vs. C2 (ref: C2)	
	OR (95% CI)	*P*	OR (95% CI)	*P*	OR (95% CI)	*P*
Education level (ref: High school and above)						
Elementary school and under	**0.068 (0.007, 0.649)**	**0.019**	**0.088 (0.009, 0.835)**	**0.034**	0.331 (0.058, 1.880)	0.212
Middle school	0.668 (0.072, 6.162)	0.722	**0.022 (0.002, 0.311)**	**0.005**	0.152 (0.023, 1.019)	0.052
Monthly income (ref: ≥5,000 RMB)						
< 1,000	**0.036 (0.011, 0.115)**	**< 0.001**	**0.011 (0.001, 0.147)**	**< 0.001**	0.314 (0.032, 3.083)	0.320
1,000~2,999	**0.094 (0.021, 0.426)**	**0.002**	**0.005 (0.001, 0.127)**	**0.001**	**0.057 (0.004, 0.922)**	**0.044**
3,000~4,999	0.560 (0.055, 5.657)	0.623	0.926 (0.029, 29.90)	0.965	1.652 (0.123, 22.186)	0.705
Health information seeking intention (ref: Willing)						
Unwilling	**0.239 (0.116, 0.492)**	**< 0.001**	**0.092 (0.030, 0.285)**	**< 0.001**	0.906 (0.257, 3.194)	0.877
Continuous variables						
CSES	**11.256 (4.849, 26.147)**	**< 0.001**	**28.919 (8.951, 93.299)**	**< 0.001**	**2.554 (1.141, 5.742)**	**0.023**
TS	**0.061 (0.021, 0.171)**	**< 0.001**	**0.072 (0.015, 0.356)**	**0.001**	**0.144 (0.027, 0.785)**	**0.025**
PSSS	**2.918 (1.480, 5.737)**	**0.002**	**3.975 (1.951, 8.050)**	**< 0.001**	1.524 (0.670, 3.426)	0.312

Bold values indicate statistical significance (*P* < 0.05). OR > 1 indicates a higher likelihood of belonging to the comparison group relative to the reference group; OR < 1 indicates a lower likelihood. C1, low literacy-restricted basic skills type; C2, moderate literacy-guided learning dependent type; C3, high literacy-independent and efficient type; For continuous variables (CSES, TS, PSSS), odds ratios (ORs) and 95% confidence intervals (CIs) are presented per 10-point increase to improve clinical interpretability; In the column headers, the group labeled ref serves as the reference category for each pairwise comparison (e.g., in C2 vs. C1, C3 vs. C1 (ref: C1), C1 is the reference; in C3 vs. C2 (ref: C2), C2 is the reference); The reference categories for independent variables were set as the highest assignment (e.g., high school and above for education, ≥ 5,000 RMB for monthly income, and willing for health information seeking intention). CSES, COPD Self-Efficacy Scale; TS, Technophobia Scale; PSSS, Perceived Social Support Scale.

Compared with the moderate literacy–guided learning dependent type (C2, reference group), patients with a monthly income of 1,000–2,999 yuan were less likely to be classified into the high literacy–independent efficient type (C3) than those with a monthly income ≥5,000 yuan (OR = 0.057, 95% CI: 0.004–0.922). Regarding continuous variables, each 10-point increase in CSES significantly increased the odds of being in C3 vs. C2 (OR = 2.554, 95% CI: 1.141–5.742), whereas each 10-point increase in TS significantly decreased the odds (OR = 0.144, 95% CI: 0.027–0.785). Education level, willingness to seek health information, and PSSS did not differ significantly between the C2 and C3 groups.

The robustness of the multivariate model was confirmed through a series of diagnostic assessments. The VIF values for all included predictors ranged from 1.02 to 1.85, indicating no significant multicollinearity. The Box-Tidwell test yielded non-significant results for all continuous variables (all *P* > 0.05 after Bonferroni correction), supporting the assumption of linearity in the logit. No influential observations were identified (maximum Cook's distance = 0.112). The model showed good overall fit (χ^2^ = 415.38, *P* < 0.001; Nagelkerke R^2^ = 0.748; the overall classification accuracy =92.5%). Additionally, the results of the sensitivity analysis conducted using Firth's penalized likelihood estimation (Table 5) were consistent with those of the primary analysis in terms of direction, effect size, and significance, confirming the robustness of the regression model. To further validate result stability against latent classification uncertainty, we conducted the R3STEP three-step sensitivity analysis. The error-adjusted R3STEP results were nearly identical to the primary classify-analyze findings across all three pairwise comparisons (C2 vs. C1, C3 vs. C1, C3 vs. C2). The full R3STEP sensitivity results are presented in Table 6. In addition to the primary stepwise model, we conducted a sensitivity analysis using the theoretically specified full model that included all univariately significant variables. The full model showed good overall fit (χ^2^ = 478.26, *P* < 0.001; Nagelkerke R^2^ = 0.782; overall classification accuracy = 88.7%). As shown in Table 7, the direction and statistical significance of all core predictors (education level, monthly income, health information seeking intention, self-efficacy, technophobia, and perceived social support) were fully consistent with the primary stepwise model, indicating that the core associations identified in this study are robust and not driven by the stepwise variable selection procedure. Other demographic and disease-related variables (e.g., age, place of residence, disease course) showed no independent significant association with DHL profile membership after simultaneous adjustment for all covariates, which was consistent with the results of the backward stepwise selection procedure.

**Table 5:** Sensitivity analysis of multifactorial latent class analysis using Firth's penalized likelihood among older adult patients with COPD.

Variables	C2 vs. C1 (ref: C1)		C3 vs. C1 (ref: C1)		C3 vs. C2 (ref: C2)	
	OR (95% CI)	*P*	OR (95% CI)	*P*	OR (95% CI)	*P*
Education level (ref: High school and above)						
Elementary school and under	**0.071 (0.009, 0.672)**	**0.021**	**0.091 (0.010, 0.862)**	**0.036**	0.338 (0.059, 1.901)	0.218
Middle school	0.672 (0.073, 6.188)	0.728	**0.023 (0.002, 0.321)**	**0.006**	0.155 (0.024, 1.035)	0.054
Monthly income (ref: ≥5,000 RMB)						
< 1,000	**0.038 (0.013, 0.122)**	**< 0.001**	**0.012 (0.001, 0.158)**	**< 0.001**	0.321 (0.034, 3.152)	0.328
1,000~2,999	**0.098 (0.022, 0.438)**	**0.002**	**0.006 (0.001, 0.132)**	**0.001**	**0.059 (0.005, 0.951)**	**0.046**
3,000~4,999	0.565 (0.057, 5.712)	0.625	0.931 (0.030, 30.050)	0.968	1.655 (0.125, 22.330)	0.708
Health information seeking intention (ref: Willing)						
Unwilling	**0.242 (0.117, 0.497)**	**< 0.001**	**0.095 (0.031, 0.291)**	**< 0.001**	0.912 (0.259, 3.215)	0.882
Continuous variables						
CSES	**11.092 (4.768, 25.766)**	**< 0.001**	**28.514 (8.811, 91.577)**	**< 0.001**	**2.498 (1.127, 5.651)**	**0.024**
TS	**0.063 (0.022, 0.175)**	**< 0.001**	**0.074 (0.016, 0.365)**	**0.001**	**0.148 (0.028, 0.800)**	**0.026**
PSSS	**2.889 (1.467, 5.700)**	**0.002**	**3.936 (1.930, 7.985)**	**< 0.001**	1.510 (0.669, 3.390)	0.318

Bold values indicate statistical significance (*P* < 0.05). OR > 1 indicates a higher likelihood of belonging to the comparison group relative to the reference group; OR < 1 indicates a lower likelihood. C1, low literacy-restricted basic skills type; C2, moderate literacy-guided learning dependent type; C3, high literacy-independent and efficient type; For continuous variables (CSES, TS, PSSS), odds ratios (ORs) and 95% confidence intervals (CIs) are presented per 10-point increase to improve clinical interpretability; In the column headers, the group labeled ref serves as the reference category for each pairwise comparison (e.g., in C2 vs. C1, C3 vs. C1 (ref: C1), C1 is the reference; in C3 vs. C2 (ref: C2), C2 is the reference); The reference categories for independent variables were set as the highest assignment (e.g., high school and above for education, ≥ 5,000 RMB for monthly income, and willing for health information seeking intention). CSES, COPD Self-Efficacy Scale; TS, Technophobia Scale; PSSS, Perceived Social Support Scale. All Firth's penalized regression analyses were implemented in R 4.4.3 using the logistf package.

**Table 6:** Sensitivity analysis of multifactorial latent class analysis using the R3STEP three-step procedure among older adult patients with COPD.

Variables	C2 vs. C1 (ref: C1)		C3 vs. C1 (ref: C1)		C3 vs. C2 (ref: C2)	
	**OR (95% CI)**	***P***	**OR (95% CI)**	***P***	**OR (95% CI)**	***P***
Education level (ref: High school and above)						
Elementary school and under	**0.070 (0.007, 0.668)**	**0.020**	**0.090 (0.009, 0.858)**	**0.036**	0.335 (0.059, 1.905)	0.217
Middle school	0.670 (0.072, 6.190)	0.725	**0.023 (0.002, 0.320)**	**0.006**	0.154 (0.023, 1.032)	0.054
Monthly income (ref: ≥5, 000 RMB)						
< 1, 000	**0.037 (0.012, 0.119)**	**< 0.001**	**0.012 (0.001, 0.156)**	**< 0.001**	0.319 (0.033, 3.125)	0.326
1, 000~2, 999	**0.096 (0.022, 0.432)**	**0.002**	**0.006 (0.001, 0.131)**	**0.001**	**0.059 (0.004, 0.941)**	**0.045**
3, 000~4, 999	0.563 (0.056, 5.685)	0.624	0.930 (0.029, 29.87)	0.967	1.658 (0.124, 22.350)	0.707
Health information seeking intention (ref: Willing)						
Unwilling	**0.241 (0.117, 0.496)**	**< 0.001**	**0.094 (0.031, 0.289)**	**< 0.001**	0.910 (0.258, 3.212)	0.880
Continuous variables						
CSES	**11.124 (4.782, 25.872)**	**< 0.001**	**28.502 (8.808, 92.301)**	**< 0.001**	**2.501 (1.125, 5.660)**	**0.024**
TS	**0.062 (0.022, 0.174)**	**< 0.001**	**0.074 (0.016, 0.363)**	**0.001**	**0.147 (0.028, 0.798)**	**0.026**
PSSS	**2.898 (1.471, 5.718)**	**0.002**	**3.932 (1.929, 8.012)**	**< 0.001**	1.512 (0.668, 3.405)	0.317

Bold values indicate statistical significance (*P* < 0.05). OR > 1 indicates a higher likelihood of belonging to the comparison group relative to the reference group; OR < 1 indicates a lower likelihood. C1, low literacy-restricted basic skills type; C2, moderate literacy-guided learning dependent type; C3, high literacy-independent and efficient type; For continuous variables (CSES, TS, PSSS), odds ratios (ORs) and 95% confidence intervals (CIs) are presented per 10-point increase to improve clinical interpretability; In the column headers, the group labeled ref serves as the reference category for each pairwise comparison (e.g., in C2 vs. C1, C3 vs. C1 (ref: C1), C1 is the reference; in C3 vs. C2 (ref: C2), C2 is the reference); The reference categories for independent variables were set as the highest assignment (e.g., high school and above for education, ≥ 5,000 RMB for monthly income, and willing for health information seeking intention). CSES, COPD Self-Efficacy Scale; TS, Technophobia Scale; PSSS, Perceived Social Support Scale.

**Table 7:** Full model sensitivity analysis: multinomial logistic regression results of factors associated with DHL profiles.

Variables	C2 vs. C1 (ref: C1)		C3 vs. C1 (ref: C1)		C3 vs. C2 (ref: C2)	
	OR (95% CI)	*P*	OR (95% CI)	*P*	OR (95% CI)	*P*
Age (ref: 60~69 years)						
70~79	0.891 (0.420, 1.889)	0.764	0.774 (0.334, 1.793)	0.547	0.869 (0.379, 1.993)	0.739
≥80	0.605 (0.229, 1.597)	0.309	0.513 (0.168, 1.567)	0.241	0.848 (0.268, 2.686)	0.779
Marital status (ref: Married)						
Unmarried/divorced/widowed	0.798 (0.419, 1.520)	0.494	0.709 (0.341, 1.475)	0.358	0.888 (0.430, 1.832)	0.747
Place of residence (ref: City)						
Rural area	0.787 (0.412, 1.504)	0.472	0.679 (0.324, 1.425)	0.308	0.863 (0.417, 1.786)	0.688
Education level (ref: High school and above)						
Elementary school and under	**0.054 (0.005, 0.562)**	**0.014**	**0.063 (0.006, 0.650)**	**0.020**	1.167 (0.111, 12.248)	0.895
Middle school	**0.221 (0.090, 0.543)**	**0.001**	**0.295 (0.107, 0.814)**	**0.018**	1.335 (0.560, 3.184)	0.511
Monthly income (ref: ≥5, 000 RMB)						
< 1, 000	**0.041 (0.012, 0.136)**	**< 0.001**	**0.013 (0.001, 0.172)**	**< 0.001**	0.338 (0.034, 3.339)	0.351
1, 000~2, 999	**0.102 (0.023, 0.461)**	**0.003**	**0.006 (0.001, 0.148)**	**0.001**	**0.062 (0.004, 0.996)**	**0.049**
3, 000~4, 999	0.601 (0.059, 6.105)	0.662	0.987 (0.030, 32.04)	0.994	1.642 (0.122, 22.05)	0.709
Medical insurance type (ref: Commercial insurance or other)						
Resident insurance	1.137 (0.592, 2.184)	0.701	0.996 (0.468, 2.119)	0.991	0.876 (0.420, 1.827)	0.722
Employee insurance	1.205 (0.601, 2.415)	0.598	1.084 (0.491, 2.393)	0.840	0.899 (0.423, 1.910)	0.784
Smoking status (ref: Quit smoking)						
Yes	1.191 (0.608, 2.333)	0.613	1.098 (0.510, 2.365)	0.808	0.922 (0.440, 1.929)	0.831
No	1.082 (0.563, 2.081)	0.813	1.130 (0.534, 2.393)	0.747	1.044 (0.501, 2.175)	0.908
Disease course of COPD (ref: >10 years)						
< 5	1.071 (0.556, 2.064)	0.837	0.942 (0.444, 1.997)	0.876	0.879 (0.424, 1.824)	0.730
5~10	0.901 (0.441, 1.840)	0.772	0.819 (0.361, 1.858)	0.629	0.909 (0.406, 2.038)	0.815
Hospitalizations within past year (ref: ≥2)						
0	0.882 (0.462, 1.685)	0.702	0.800 (0.382, 1.676)	0.554	0.907 (0.442, 1.863)	0.791
1	0.724 (0.344, 1.524)	0.393	0.660 (0.279, 1.562)	0.344	0.912 (0.387, 2.147)	0.833
GOLD Classification (ref: IV)						
I	1.215 (0.547, 2.699)	0.633	1.372 (0.559, 3.368)	0.493	1.129 (0.472, 2.702)	0.787
II	1.142 (0.573, 2.274)	0.706	1.189 (0.533, 2.653)	0.674	1.041 (0.483, 2.245)	0.920
III	0.904 (0.446, 1.833)	0.780	0.798 (0.350, 1.820)	0.590	0.883 (0.396, 1.970)	0.761
Frequency of internet use (ref: Always)						
Seldom	0.647 (0.299, 1.401)	0.271	0.514 (0.205, 1.288)	0.152	0.794 (0.322, 1.958)	0.615
Sometimes	0.822 (0.397, 1.702)	0.595	0.709 (0.305, 1.649)	0.423	0.863 (0.380, 1.960)	0.722
Health information seeking intention (ref: Willing)						
Unwilling	**0.248 (0.117, 0.523)**	**< 0.001**	**0.096 (0.030, 0.300)**	**< 0.001**	0.387 (0.119, 1.257)	0.113
Query skill (ref: Can complete independently)						
Unable to complete	0.700 (0.318, 1.540)	0.376	0.582 (0.227, 1.493)	0.260	0.831 (0.325, 2.126)	0.697
Can complete with assistance	0.847 (0.432, 1.661)	0.628	0.778 (0.361, 1.679)	0.523	0.919 (0.436, 1.934)	0.825
Health software usage (ref: Yes)						
No	0.892 (0.455, 1.747)	0.736	0.768 (0.353, 1.672)	0.506	0.861 (0.405, 0.830)	0.695
Continuous variables (per 10-point increase)						
CSES	**10.924 (4.542, 26.263)**	**< 0.001**	**27.761 (8.252, 93.410)**	**< 0.001**	**2.541 (1.103, 5.856)**	**0.029**
TS	**0.064 (0.022, 0.189)**	**< 0.001**	**0.075 (0.015, 0.376)**	**0.002**	**0.170 (0.029, 0.987)**	**0.048**
PSSS	**2.847 (1.398, 5.799)**	**0.004**	**3.908 (1.832, 8.337)**	**< 0.001**	1.373 (0.722, 2.610)	0.332

Bold values indicate statistical significance (*P* < 0.05). OR > 1 indicates a higher likelihood of belonging to the comparison group relative to the reference group; OR < 1 indicates a lower likelihood. C1, low literacy-restricted basic skills type; C2, moderate literacy-guided learning dependent type; C3, high literacy-independent and efficient type; For continuous variables (CSES, TS, PSSS), odds ratios (ORs) and 95% confidence intervals (CIs) are presented per 10-point increase to improve clinical interpretability; In the column headers, the group labeled ref serves as the reference category for each pairwise comparison (e.g., in C2 vs. C1, C3 vs. C1 (ref: C1), C1 is the reference; in C3 vs. C2 (ref: C2), C2 is the reference); Model fit: χ^2^ = 478.26, *P* < 0.001; Nagelkerke R^2^ = 0.782; overall classification accuracy = 88.7%. CSES, COPD Self-Efficacy Scale; TS, Technophobia Scale; PSSS, Perceived Social Support Scale.

It should be noted that the large odds ratios observed for some continuous variables (e.g., CSES) and their wide confidence intervals should be interpreted with caution. These estimates reflect the 10-point increment scaling used in the analysis; given that the CSES total score ranges from 31 to 155, a 10-point increase represents a substantial change in self-efficacy. The wide confidence intervals may also reflect relatively small sample sizes within certain latent classes and the inherent uncertainty in estimating effects for rare category memberships. Nevertheless, the direction and significance of these associations were consistently confirmed across multiple sensitivity analyses, supporting the robustness of our findings.

## 4 Discussion

### 4.1 Three latent profiles of digital health literacy in older adult patients with COPD

Health literacy—defined as the capacity to obtain, process, and understand basic health information and services and to make appropriate health decisions based on such information—has long been recognized as a critical determinant of health outcomes and a major challenge for healthcare systems worldwide (30). Surveys indicate that limited or inadequate health literacy is particularly prevalent among older adults and individuals with chronic conditions, placing a substantial burden on public health resources (31). In the contemporary digital era, this foundational challenge has evolved into a new imperative: DHL. As healthcare services increasingly migrate online, the ability to navigate, evaluate, and apply digital health information has become a prerequisite for effective self-management. Notably, the prevalence of limited DHL observed in our study does not markedly diverge from the broader health literacy statistics (31). This finding suggests that the barriers to understanding and using health information, whether in traditional or digital formats, remain pervasive and may share common underlying factors, such as socioeconomic status, cognitive ability, and access to educational resources. The parallel between general and DHL underscores that the digital divide is not merely a technological issue but a profound extension of existing health inequities.

To our knowledge, this represents the first comprehensive examination of the heterogeneity in DHL among older adult patients with COPD. Further analysis was conducted to evaluate the associations of sociodemographic characteristics, health information seeking intention, self-efficacy, technophobia, and social support across distinct literacy subgroups. Significant heterogeneity in DHL was identified within this population. On the basis of the results obtained from model fitting, we categorize them into three latent groups: low literacy-restricted basic skills type, moderate literacy-guided learning dependent type, and high literacy-independent and efficient type.

C1 comprising 37.1% of the overall sample, demonstrated the lowest scores on each of the seven dimensions of DHL, indicating a generally low level of competency within this subgroup. Furthermore, this group demonstrated comparatively weaker social support and self-efficacy, along with higher technology-related anxiety, which collectively eroded confidence in using digital health tools (32). This finding aligns with prior evidence suggesting that multiple barriers can impede the effective improvement of patients' digital health literacy (33). Consequently, it is suggested that healthcare providers should prioritize this vulnerable subgroup and deliver strengthened, targeted support.

C2 accounted for 28.9% of the total sample and demonstrated a moderate level of overall DHL. Analysis of characteristics indicated that this group scored relatively low in navigation skills, information reliability assessment, and privacy protection, whereas high confidence was observed in operational skills. This suggests that despite possessing basic operational capabilities and self-assurance, their capacity for information evaluation and integration remains underdeveloped, On the one hand, while patients have accumulated some practical experience through daily exposure, they lack systematic digital health training. Consequently, they often struggle to make effective judgments when discerning the authenticity of online health information. On the other hand, this may be linked to patients' relatively lower income and educational levels (34). Consequently, interventions targeting this group should prioritize converting confidence into actionable practical skills, thereby promoting a transition from passive dependency to proactive coping.

C3 accounted for 34.0% of the total sample. This group was characterized by significantly higher DHL across all assessed dimensions relative to the other groups. Notably, they exhibited strong operational skills, indicating a robust capacity for searching for and applying health information. Further analysis revealed that this group possessed richer psychosocial resources, such as social support and self-efficacy, which played a pivotal role in enhancing DHL. Specifically, social support bolstered their confidence and capability in using digital tools by providing informational resources, emotional encouragement, and practical assistance. Meanwhile, self-efficacy motivated them to proactively learn and apply digital health technologies, thereby demonstrating greater initiative and effectiveness in disease management. These psychosocial resources collectively fostered the advancement of DHL (35). Therefore, future interventions could capitalize on this group's high levels of self-efficacy and social support, distilling their experiences of proactively learning and applying digital tools into a set of replicable methods. This would assist other patients with relatively fewer resources in enhancing their DHL amidst adversity. Overall, these results are consistent with recent LPA studies focusing on older adult populations with chronic diseases in China. For instance, Luo et al. identified three DHL profiles (low, medium, and high) among patients with coronary heart disease, and these profiles closely resemble our observations (36). Shao et al. also reported a similar three-profile pattern in their DHL research on older adult patients with chronic diseases (37).

### 4.2 Factors associated with the latent profile of digital health literacy

#### 4.2.1 Individual level: economic and educational foundations

This study demonstrates that both educational level and monthly income are significantly associated with DHL profile membership, consistent with previous research (37, 38). Compared with the low literacy-restricted basic skills type, individuals with higher education levels were more frequently classified into the high literacy-independent and efficient or moderate literacy-guided learning dependent types. Similarly, patients with higher incomes were more likely to be categorized as either high literacy-independent and efficient or moderate literacy-guided learning dependent types. These findings reflect the cumulative advantage framework, wherein socioeconomic resources enable earlier adoption of digital technologies, greater access to digital health services, and more extensive social and technical support (39). Higher education is generally associated with superior health literacy, which enables more effective access, comprehension, and application of health-related information (40). Conversely, patients with lower educational levels often experience greater difficulty accessing and processing health-related information and struggle to systematically acquire disease knowledge or information retrieval skills (32). Such challenges frequently lead to confusion and uncertainty when digital health platforms or resources are used, subsequently reducing willingness to engage with online health information.

The interaction between education and income is particularly noteworthy. Low-income older adults often face challenging living conditions and lack stable income sources. Due to significant financial pressures, they may lack both the inclination and capacity to proactively seek online health information, tending instead toward simple self-treatment approaches to manage health issues (41). These findings suggest that older adults with low socioeconomic status may represent a high-risk population with reduced DHL, warranting greater attention in subsequent research and policy development. From a clinical practice perspective, the following approaches could be considered to support DHL improvement among less-educated and lower-income patients, though their effectiveness has not been tested in this study: (1) use of plain language and visual aids to improve comprehension; (2) provision of free digital health services; (3) streamlined access to information; and (4) assistance in more effectively accessing, comprehending, and utilizing online health information.

#### 4.2.2 Behavioral and psychological characteristics level: psychological and motivational mechanisms

DHL is closely linked to factors within the psychological dimension, including self-efficacy, technophobia and willingness to seek health information. Our findings demonstrate that individuals with lower self-efficacy and higher technophobia are more likely to be classified into the low literacy-restricted basic skills type, consistent with previous research (37, 42). This association may be explained by the potential interplay between self-efficacy and technophobia: higher self-efficacy enables more proactive engagement with disease-related challenges and better maintenance of long-term healthy self-management behaviors (43), while lower self-efficacy is frequently associated with negative attitudes toward digital health tools, failure to sustain consistent health monitoring, and reduced participation in self-management programmes (44). Technophobia further diminishes individuals' perception of the ease of use of digital health technologies, correlating with technology avoidance behaviors that restrict actual use (45). Although these theoretical pathways suggest a relationship, it is important to note that the cross-sectional design of this study precludes formal tests of mediation or causal inference. In contrast, individuals with lower levels of technophobia tend to be more willing to actively explore digital health information, adapt more quickly to mobile devices, and report more positive user experiences (46).

Health information seeking intention acts as a potential driver and a consequence of these psychological factors and is strongly associated with DHL levels. Patients exhibiting a higher propensity for information seeking are more likely to be categorized as either high literacy-independent and efficient or moderate literacy-guided learning dependent types. This may reflect that such information-seeking inclination directly reflects acceptance of digital technologies and has a positive impact on the improvement of DHL (47). Conversely, a lower propensity for information-seeking directly limits older adults' access to and frequency of engagement with digital health resources, hindering the effective development and enhancement of digital skills through practical application (48). This observed association potentially creates a self-reinforcing cycle: low self-efficacy and high technophobia are associated with reduced information seeking, which in turn may limit skill development, further reinforcing low confidence and anxiety. However, this proposed cyclical pathway remains speculative and requires longitudinal evidence for confirmation. Future interventional programs could potentially address these psychological barriers through the following approaches, which remain to be empirically validated: (1) active reinforcement and sharing success stories to enhance self-efficacy; (2) employing diverse channels and formats to help older adults understand and master digital technologies, thereby reducing technophobia; (3) lowering barriers and offering proactive guidance for patients exhibiting low information-seeking tendencies.

Notably, these internet-related behavioral and intentional variables are conceptually distinct from the DHL competency construct used to define the latent profiles. DHL reflects an individual's intrinsic, multi-dimensional ability to navigate digital health resources, whereas internet use frequency, basic query skill, information seeking intention and health software use represent external behavioral manifestations, motivational drivers and foundational practice conditions for literacy development. From a developmental perspective, higher use frequency, stronger information-seeking intention, better basic query skills and more hands-on experience with health software provide more opportunities for skill practice and knowledge accumulation, which in turn promote the improvement of health-specific digital literacy. This follows a motivation-practice-competency developmental pathway, explaining these factors are strongly associated with DHL profile membership.

#### 4.2.3 Interpersonal network level: social support and family networks

Research findings indicate that patients with higher social support scores are more likely to be classified into either the high literacy-independent and efficient or moderate literacy-guided learning dependent types, compared with those in the low literacy-restricted basic skills type, consistent with previous research (37). This association is attributed to the reinforcing effect of strong social support on patients' confidence and perceived control over disease management (49). Moreover, as the most critical and pervasive source of support, the family is central to daily care, symptom monitoring, and health decision-making (50).

However, the translation of available social support into effective care delivery faces substantial challenges in the Chinese context. Primary healthcare institutions face constraints such as insufficient staffing and inadequate facilities, which hinder the translation of available social support into effective care delivery (51). Consequently, the capacity of these institutions to meet patients' diverse support needs remains compromised. For future nursing practice, the following directions could be prioritized for exploration and evaluation: (1) harnessing family-based resources and actively engaging relatives in the health management process; (2) strengthening the delivery capacity of primary care settings; (3) ensuring a more integrated and supportive healthcare framework; and (4) improving service quality to better fulfill the varied support requirements arising during COPD patient management.

Furthermore, our results indicate that routine universal offering of digital health interventions to all COPD patients may not be appropriate. Patients with GOLD IV classification demonstrated significantly lower DHL scores, which may reflect the combined burden of severe physical limitations (e.g., dyspnea limiting device interaction), cognitive challenges associated with chronic hypoxemia, and reduced energy for learning new technologies. For these patients, digital health interventions could inadvertently exacerbate health disparities by diverting resources away from more appropriate, low-technology interventions that better accommodate their functional limitations. This aligns with recent guidance suggesting that digital health interventions should be offered based on patient capability and preference rather than being universally applied (52). Based on our findings, the following clinical practices could be considered for future implementation and evaluation: (1) systematic screening for DHL and physical/cognitive capacity prior to initiating digital health interventions; (2) maintaining a portfolio of low-technology self-management support options for patients with GOLD IV or severe functional limitations; (3) offering a stepped-care hybrid approach that combines simplified digital tasks with intensive human facilitation, with support potentially tapered as patient competence improves. These proposed practices remain untested in the current study and require prospective evaluation to confirm their real-world utility and effectiveness.

## 5 Limitations

Several constraints of this research warrant acknowledgment. First, the cross-sectional survey design can only reveal correlations between variables, without enabling inferences about their dynamic changes or establishment of causality across time. Consequently, while we discuss potential pathways such as the interplay between self-efficacy, technophobia, and DHL, we cannot draw formal conclusions regarding mediation or the direction of these relationships Second, this study adopted a single-center design with hospital-based recruitment. Participants were consecutively enrolled via convenience sampling—a form of nonprobability sampling—from the respiratory medicine outpatient clinic and inpatient wards of a single Grade A tertiary hospital in Anhui Province. This non-probability sampling approach carries inherent selection bias, and the hospital-based recruitment strategy means the sample may not represent older adults with COPD who do not seek hospital-based care, receive treatment in primary care settings, or live in long-term care facilities. Coupled with the limited geographic coverage of the study site, these factors result in limited generalizability of the findings to the broader population of older adults with COPD across other regions of China or different healthcare delivery contexts. In addition, relatively small cell sizes for some categorical variables within certain latent classes may introduce sparse-data bias and slightly reduce the precision of effect size estimates. Although we validated the robustness of the results using Firth's penalized likelihood estimation to mitigate sparse-data bias, future studies with larger sample sizes or refined stratified sampling designs are still recommended to produce more stable parameter estimates. The 25 excluded questionnaires with uniform responses across all items may reflect limited reading proficiency or comprehension difficulties in some participants, which could introduce additional selection bias. Furthermore, the exclusion of the objective assessment component of the DHL instrument was a *post-hoc* decision rather than a prespecified one. Although this decision was based on substantial feasibility challenges encountered during administration and the low reliability reported in the validation study, we acknowledge that *post-hoc* exclusions may introduce additional bias and reduce the comprehensiveness of the DHL measurement. Future studies should consider adapting or simplifying the objective component for older populations rather than excluding it entirely. Third, all data were collected using self-reported questionnaires, which are susceptible to recall bias and social desirability bias that may affect the accuracy of responses. Furthermore, for participants unable to complete the questionnaire independently, the survey was administered through one-to-one oral interviews conducted by research staff. This mode of administration introduces possible interviewer bias, as variations in questioning style, item explanation, or prompting across different investigators may systematically influence participant responses. Fourth, classification uncertainty is an intrinsic limitation of latent profile analysis. Although the final three-profile solution demonstrated excellent classification performance (Entropy = 0.995), and sensitivity analyses using both the R3STEP three-step procedure and Firth's penalized likelihood estimation confirmed the robustness of covariate effect estimates, the identified profiles are statistically derived latent subgroups rather than discrete, naturally occurring categories. Therefore, the boundaries between profiles should be interpreted with appropriate caution. In addition, the backward stepwise variable selection approach used in the primary analysis carries a risk of overfitting and inflated Type I error. Although we verified the robustness of the core findings through a theoretically specified full model sensitivity analysis, future studies with larger, representative samples are still needed to validate the final predictor set and generalizability of the model. Moreover, the large odds ratios and wide confidence intervals observed for some continuous predictors should be interpreted cautiously, as they may partly reflect the 10-point increment scaling and the limited sample size within specific latent classes. While the consistent results from sensitivity analyses support the robustness of our findings, the absolute magnitude of these ORs should not be overinterpreted as indicating deterministic risk, but rather as reflecting the strong association between psychological resources and DHL levels in this population. Future studies with larger, more diverse samples are needed to obtain more precise and stable effect estimates. Fifth, residual confounding remains a notable limitation of this study. Several potentially relevant factors were not measured and thus could not be adjusted for in the analysis. For instance, general health literacy—defined as the capacity to obtain, process, and understand basic health information—may act as a foundational competency underlying DHL, but it was not included as a covariate. Among older adults with COPD, barriers to digital engagement appear to be more prominent in terms of technical confidence and psychological readiness, which may limit the degree to which general health literacy translates into functional DHL. The exact nature of this relationship therefore remains unclear and may represent an unmeasured confounder. Additionally, this study did not collect individual-level data on air pollution exposure or asthma comorbidity, which restricts further exploration of how non-smoking-related COPD phenotypes interact with digital health literacy and constitutes another source of residual confounding. Future research incorporating general health literacy, detailed environmental exposure data, and comprehensive clinical phenotype information will help clarify these associations.

## 6 Conclusion

This study utilized LPA to explore the heterogeneous nature of DHL among older adults diagnosed with COPD. Three distinct latent profiles were identified: low literacy-restricted basic skills type, moderate literacy-guided learning dependent type, and high literacy-independent and efficient type. Furthermore, several factors were found to be associated with profile membership, including educational level, monthly income, willingness to use health information, self-efficacy, technophobia and perceived social support. Based on these observational findings, personalized, multi-level intervention strategies could be hypothetically developed by integrating the characteristics of each subtype and their associated factors, grounded in the health ecology framework. These targeted strategies may have the potential to enhance the DHL of this population and support their long-term self-management, but their actual effectiveness and clinical utility require dedicated interventional studies to verify.

## Data Availability

The datasets presented in this article are not readily available because the datasets generated and analyzed during the current study are not publicly available, but are available from the corresponding author on reasonable request. Requests to access the datasets should be directed to MB: efy2013101@fy.ahmu.edu.cn.
